# Impact of Drying Processes on the Nutritional Composition, Volatile Profile, Phytochemical Content and Bioactivity of *Salicornia ramosissima* J. Woods

**DOI:** 10.3390/antiox10081312

**Published:** 2021-08-20

**Authors:** Sheila C. Oliveira-Alves, Fábio Andrade, Inês Prazeres, Andreia B. Silva, Jorge Capelo, Bernardo Duarte, Isabel Caçador, Júlio Coelho, Ana Teresa Serra, Maria R. Bronze

**Affiliations:** 1 iBET, Instituto de Biologia Experimental e Tecnológica, Apartado 12, 2781-901 Oeiras, Portugal; sheila.alves@ibet.pt (S.C.O.-A.); f.andrade@campus.ul.pt (F.A.); ines.prazeres@ibet.pt (I.P.); tserra@ibet.pt (A.T.S.); 2 DCFM, Departamento de Ciências Farmacêuticas e do Medicamento, Faculdade de Farmácia, Universidade de Lisboa, Av. das Forças Armadas, 1649-003 Lisboa, Portugal; abentosilva@ff.ulisboa.pt; 3 iMed ULisboa, Instituto de Investigação do Medicamento, Faculdade de Farmácia, Universidade de Lisboa, Av. Prof. Gama Pinto, 1649-003 Lisboa, Portugal; 4 INIAV, Instituto Nacional de Investigação Agrária e Veterinária, Av. da República, 2780-505 Oeiras, Portugal; jorge.capelo@iniav.pt; 5 MARE, Marine and Environmental Sciences Centre, Faculdade de Ciências, Universidade de Lisboa, Campo Grande, 1749-016 Lisboa, Portugal; baduarte@fc.ul.pt (B.D.); micacador@fc.ul.pt (I.C.); 6 Departamento de Biologia Vegetal, Faculdade de Ciências, Universidade de Lisboa, Campo Grande, 749-016 Lisboa, Portugal; 7 Horta da Ria Lda., Rua de São Rui, 3830-362 Gafanha Nazaré, Portugal; geral@hortadaria.pt; 8 Instituto de Tecnologia Química e Biológica António Xavier, Universidade Nova de Lisboa, Av. da República, 2780-157 Oeiras, Portugal

**Keywords:** flavonoids, antioxidants, minerals, volatile compounds, hypertension

## Abstract

*Salicornia ramosissima* J. Woods is a halophyte plant recognized as a promising natural ingredient and will eventually be recognized a salt substitute (NaCl). However, its shelf-life and applicability in several food matrices requires the use of drying processes, which may have an impact on its nutritional and functional value. The objective of this study was to evaluate the effect of oven and freeze-drying processes on the nutritional composition, volatile profile, phytochemical content, and bioactivity of *S. ramosissima* using several analytical tools (LC-DAD-ESI-MS/MS and SPME-GC-MS) and bioactivity assays (ORAC, HOSC, and ACE inhibition and antiproliferative effect on HT29 cells). Overall, results show that the drying process changes the chemical composition of the plant. When compared to freeze-drying, the oven-drying process had a lower impact on the nutritional composition but the phytochemical content and antioxidant capacity were significantly reduced. Despite this, oven-dried and freeze-dried samples demonstrated similar antiproliferative (17.56 mg/mL and 17.24 mg/mL, respectively) and antihypertensive (24.56 mg/mL and 18.96 mg/mL, respectively) activities. The volatile composition was also affected when comparing fresh and dried plants and between both drying processes: while for the freeze-dried sample, terpenes corresponded to 57% of the total peak area, a decrease to 17% was observed for the oven-dried sample. The oven-dried *S. ramosissima* was selected to formulate a ketchup and the product formulated with 2.2% (*w/w*) of the oven-dried plant showed a good consumer acceptance score. These findings support the use of dried *S. ramosissima* as a promising functional ingredient that can eventually replace the use of salt.

## 1. Introduction

Halophyte plants are naturally evolved salt-tolerant plants commonly found in saltmarshes and coastal areas worldwide, representing at most 2% of terrestrial plant species [1,2]. Among steam succulent halophytes, *Salicornia* species are evolved into physiological forms that allow them to cope with excessive salinity, high temperature amplitudes, and elevated irradiances through the production of osmoprotective, antioxidant, and photoprotective metabolites [3,4], making them ideal candidates for bioprospection of added-value compounds.

The annual edible halophyte *Salicornia ramosissima* J. Woods, also known as glasswort and sea asparagus, belongs to the *Amaranthaceae* family and is widespread on the saltmarshes of the Iberian Peninsula [5,6]. This specie has been introduced into the European market as a vegetable with leafless shoots due to its slight salty taste, which is appreciated in gourmet cuisine as a promising salt substitute [7,8,9].

Nutritionally, *S. ramosissima* is a source of proteins, fibers, minerals, and polyunsaturated fatty acids. Among phytochemicals, phenolic compounds [7,10] such as quercetin-3-*O*-glucoside and caffeoylquinic acids were already identified in *S. ramosissima*. These compounds are known to play an important role in the prevention of different disorders such as cancer, hypertension, and cardiovascular diseases [11,12,13,14]. Studies state that the phenolic compounds from *S. ramosissima* display antioxidant activity and a photoprotective effect against UV radiation [15], and have a therapeutic value on the male reproductive system [16].

Currently, *S. ramosissima* has been recognized as a promising candidate for the food industry through its applicability as a natural ingredient for the development of new products with functional and beneficial health properties [17,18,19,20]. However, fresh *S. ramosissima* is sensitive to microbial spoilage even when refrigerated, thus necessary to freeze or dry to extend its shelf-life [21]. The agroindustry has applied the oven-drying process, one of the most widely used preservation methods, in genus *Salicornia* as the preservation method to allow for its use as a condiment or ingredient in foods. However, the impact on the nutritional and phytochemical content after the drying process of fresh *S. ramosissima* has not been investigated. According to a study that applied a convective drying process between 40 and 70 °C to *Sarcocornia perennis*, the drying at 70 °C proved to be the most appropriate methodology to preserve its health-beneficial properties [21].

The present study aimed to evaluate the effect of two drying processes (oven and freeze-drying) on the quality attributes and bioactivity of *S. ramosissima*. Several analytical methods, enzymatic assays, and cell-based studies were used to compare the nutritional content, phenolic composition, antioxidant activity, antihypertensive effect, and antiproliferative capacity of the dried plants. For the first time, the volatile composition of this plant (fresh and dried) was described and its acceptance was evaluated after formulation in a food product.

## 2. Materials and Methods

### 2.1. Halophyte Plant

Fresh and oven-dried *Salicornia ramosissima* J. Woods were obtained from Horta da Ria Lda. (Aveiro, Portugal). The fresh plants were collected in high saltmarsh between July and October 2019 in the region of Aveiro (north of Portugal). Upon reception, a portion of fresh plants was stored in plastic bags at 7 °C until its volatile, nutritional, and phytochemical analyses were performed. Another portion of fresh plants was stored in plastic bags at −20 °C for subsequent freeze-drying. Taxonomic identity of specimens was based on morphological and morphometric features following the criteria of Valdés and Castroviejo (1990) [22].

### 2.2. Drying Process

The freeze-drying process of the *S. ramosissima* was carried out by first grinding (Grinder Mill Flama, Aveiro, Portugal) the whole fresh frozen plants in liquid nitrogen (fast freezing) and then lyophilizing (ScanVac, Coolsafe 95/55–80 freeze dryer, Lynge, Denmark) for 24 h (moisture < 3.5%). According to the producer, the oven-dried *S. ramosissima* production was accomplished by placing the whole fresh plants into a dry oven at 70 °C for 72 h. After drying, the whole oven-dried plants were packed in colorless plastic bags and sent to the laboratory. In the laboratory, the whole oven-dried plant was ground using a mill (Cyclone mill, Retsch Twister, Haan, Germany), homogenized, and packed in amber glass bottles. The oven-dried and freeze-dried *S. ramosissima* were stored in amber glass bottles at −20 °C until analyses.

### 2.3. Reagents

Acetonitrile (CH_3_CN, 99.9% LC–MS) and methanol (MeOH, 99.8% LC–MS) were purchased from Fisher Scientific (Thermo Fisher Scientific, Waltham, MA, USA). The formic acid (HCOOH, 98% p.a.) was purchased from Merck (Darmstadt, Germany). The ultra-pure water (18.2 MO.cm) was obtained from a Millipore-Direct Q3 UV system (Millipore, Burlington, MA, USA). Fluorescein sodium salt, Folin–Ciocalteu’s reagent Trolox (6-hydroxy-2,5,7,8-tetramethyl chromane-2-carboxylic acid) and AAPH (2,2-azobis(2-methylpropionamidine)dihydrochloride), were all obtained from Sigma-Aldrich (Darmstadt, Germany). Standard phenolic compounds, namely gallic acid (PubChem CID: 370) and quercetin-3-glucoside (PubChem CID: 25203368), were obtained from Sigma-Aldrich (Darmstadt, Germany), and chlorogenic acid (PubChem CID: 1794427) and quercetin-3-*O*-(6-acetylglucoside) were obtained from Extrasynthese (Genay, France). MTS reagent (CellTiter 96^®^ AQueous One Solution Reagent) was obtained from Promega (Madison, WI, USA).

### 2.4. Nutritional Characterization

#### 2.4.1. Nutritional Parameters

Moisture was determined in an oven-drying at 105 ± 1 °C and ash content was quantified by means of sample incineration in a muffle furnace (600 °C) as described by the Association of Official Analytical Chemists [23]. Total protein was determined by the Kjeldahl method (F = 6.25) and total fat using the Soxhlet extraction method [23]. Total energy value was calculated according to the European Regulation 1169/2011 (European Parliament and Council of the European Union, 2006). Total dietary fiber was determined according to the AOAC official method 985.29 [23]. The fatty acids composition of the sample was analyzed by gas chromatography (GC) with a flame ionization detector (FID). The fatty acids methyl esters (FAMEs) were performed as described by the AOCS method Ce 2–66 [23]. FAMEs were identified by comparison of retention times with FAMEs standard mixture under the same conditions (FAME Mix C4-C24 Sulpeco, Bellefonte, PA, USA) and quantified using area normalization. Carbohydrates were calculated by difference using Equation (1). All the measurements were performed in triplicates for each analysis.

[Carbohydrates = 100 − (Ash + Moisture + Protein + Total fat)] (1)

#### 2.4.2. Mineral Composition

The mineral composition of fresh *S. ramosissima* was determined by Flame Atomic Absorption Spectrometry (FAAS) [23]. Minerals of dried *S. ramosissima* were extracted by the digestion process and quantified using total X-ray fluorescence spectroscopy (TXRF) element analysis [24]. This procedure of digestion was carried out in Teflon reactors: 200 mg of the sample were weighted into digestion reactors and 0.3 mL HClO_4_ and 1.7 mL HNO_3_ were added. The reactors were tightly closed and digestion occurred during 3 h in an oven at 110 °C [25]. After cooling, for each sample, 496 µL were recovered into 1.5 mL tubes and 2 µL of Ga (final concentration 1 mg L^−1^) was added as the internal standard for mineral quantification, spiked with 2 µL Cd (final concentration 1 mg L^−1^) [26]. Samples were stored at 4 °C until analysis. Mineral quantification was determined through total X-ray fluorescence spectroscopy (TXRF) element analysis. The cleaning and preparation of the TXRF quartz glass sample carriers were performed and 5 µL of the digested sample was added to the center of the carrier [26]. The carriers with the samples were aligned in a support containing a carrier with arsenic for gain correction (mono-element standard, Bruker Nano GmbH, Karlsruhe, Germany), a carrier with a nickel standard for sensitivity and detection limit (mono-element standard, Bruker Nano GmbH, Karlsruhe, Germany), and a carrier with a multi-element kraft for quantification accuracy (Kraft 10, Bruker Nano GmbH, Karlsruhe, Germany). Element measurements were carried out in a portable benchtop TXRF instrument (S2 PICOFOX™ spectrometer, Bruker Nano GmbH) for 800 s per sample. The accuracy and precision of the analytical methodology for elemental determinations were assessed by replicate analysis of certified reference material (BCR-146). Blanks and the concurrent analysis of the standard reference material were used to detect possible losses/contamination during analysis. The TXRF spectra and data evaluation interpretation were accomplished using the Spectra 7.8.2.0 software (Bruker Nano GmbH, Berlin, Germany).

### 2.5. Volatile Composition by Gas Chromatography

Solid phase microextraction (SPME) was used for the analysis of volatile compounds from fresh and dried plants. Briefly, the fresh *S. ramosissima* was crushed with a mortar and pestle until a paste was formed, after which 1.5 g of paste was transferred to a GC-MS vial. For the dried plants, 0.5 g was transferred directly to a GC-MS vial. A 20 mL headspace vial (La-Pha-Pack^®^, Langerwehe, Germany) capped with a white PTFE silicone septum (Specanalitica, Carcavelos, Portugal) was used in both situations. The SPME operating conditions consisted of an extraction temperature of 40 °C for 40 min, a rotating speed of 250 rpm, an agitation of 10 s, and desorption time of 3 min at 250 °C. Analyses were performed in a GCMS QP2010 Plus (Shimadzu, Kyoto, Japan) equipped with an AOC-5000 autosampler (Shimadzu^®^). For headspace, SPME sampling used a divinylbenzene/carboxen/polydimethylsiloxane (DVB/Car/PDMS) fiber (Supelco Analytical, Bellefonte, PA, USA). For the separation of volatile compounds, a capillary column Sapiens-5-MS (Teknokroma, Barcelona, Spain) with dimensions of 30 m, 0.25 mm (IS), and 0.25 μm (film thickness) was used. The working conditions were as follows. The injector and detector temperature were maintained at 250 °C. The injection mode was carried out in the splitless mode during 1.5 min and high-purity helium (≥99.999%) was used as the carrier gas. The column oven temperature was kept at an initial temperature of 40 °C for 5 min, increased to 170 °C at a rate of 5 °C min^−1^, and then was increased to 230 °C at 30 °C min^−1^ and maintained for 4 min; carrier gas (He) was maintained with a flow of 2.00 mL min^−1^. In the MS interface, the temperature was 250 °C and the ion source temperature was 250 °C. Mass spectra were acquired in the electron ionization (EI) mode at 70 eV in a *m*/*z* range between 29 and 300 with a scan speed of 588 scans s^−1^. Identification of compounds was performed using the mass spectra library (NIST 2005 mass spectra database, Boulder, CO, USA) and Linear Retention Index (RI) [27,28]. Each sample was analyzed in duplicates.

### 2.6. Analysis of Phenolic Composition

#### 2.6.1. Extraction of Phenolic Compounds

The extraction of phenolic compounds was performed using an ultrasound extraction procedure (USE) [18,19]. Briefly, the fresh plant was crushed with a mortar and pestle with the addition of liquid nitrogen, whereas the dried plant was ground in a cyclone mill (Retsch Twister). The extractions were performed by adding 100 mL of ethanol:water (80:20, *v*/*v*) solution to 10 g of the fresh plant or 2 g of the dried plant, respectively. The samples were placed in the vortex for 10 s and then immediately transferred to an ultrasonic water bath (ArgoLab DU-100, Carpi, Modena, Italy) at 40 kHz and 220 W for 60 min at 25 ± 3 °C. The samples were then centrifuged at 6000× *g* for 15 min (Sorvall ST16 centrifuge, Thermo Scientific, Osterode, Germany) and the supernatant removed. The supernatant was evaporated to dryness at 40 ± 1 °C under reduced pressure (120 Bar) using a rotavapor (Büchi R-114, Flawil, Switzerland). The dried residue was dissolved in 2 mL of ethanol:water (50:50, *v*/*v*) solution, filtered through a 0.22 mm SFCA membrane Branchia (Labbox Labware, Barcelona, Spain), and stored at −18 °C until analysis. All extractions were carried out in triplicates.

#### 2.6.2. HPLC-DAD-ESI-MS/MS

In order to characterize the phenolic composition, the extracts were analyzed in a Waters Alliance 2695 HPLC system (Waters, Milford, MA, USA) equipped with a quaternary pump, solvent degasser, auto sampler, and column oven, coupled to a diode array detector (DAD), namely the Detector Waters 2996 (Waters, Milford, MA, USA). For the separation of compounds, a pre-column (100RP-18, 5 mm) and reversed phase C18 column (LiCrospher 100 RP-18, 250 × 4 mm; 5 mm) in a thermostatic oven at 35 °C were used. The mobile phase consisted of water:formic acid (99.5%:0.5%) as eluent A and acetonitrile:formic acid (99.5%:0.5%) as eluent B at a flow rate of 0.30 mL/min. All solvents were filtered through a 0.22 mm PVDF membrane (Millipore, Billerica, MA, USA) prior to analysis. The system was run with the following gradient elution program: 0–10 min from 99 to 95% A; 10–30 min from 95 to 82% A; 30–44 min from 82 to 64% A; 44–64 min at 64% A; 64–90 min from 64 to 10% A; 90–100 min at 10% A; 100–101 min from 10 to 95% A; 101–120 min at 95% A; and finally returning to the initial conditions. The injection volume was 20 µL. DAD was used to scan the wavelength absorption from 200 to 650 nm. Tandem mass spectrometry (MS/MS) detection was performed using an electrospray ionization source (ESI) at 120 °C and applying a capillary voltage of 2.5 kV and cone voltage of 30 V. The compounds were ionized in the negative mode and spectra were recorded in the range of *m*/*z* 60–1500. Analytical conditions were optimized to maximize the precursor ion signal ([M−H]^−^). Ultra-high purity argon (Ar) was used as a collision gas. High purity nitrogen (N_2_) was used both as a drying gas and nebulizing gas. For data acquisition and processing, MassLynx software (version 4.1,Waters Corporation, Milford, MA, USA) was used.

#### 2.6.3. HPLC-DAD

The phenolic compounds were quantified using a UHPLC Vanquish (Thermo Fisher Scientific, Waltham, MA, USA) equipped with an auto-sampler, pump, and Vanquish diode array detector (DAD). The chromatographic separation of compounds was carried on a Luna C18 reversed phase (Luna 5 µm C18 (2) 100 Å, 250 × 4 mm; Phenomenex) and a Manu-cart RP-18 pre-column in a thermostatic oven at 35 °C. DAD was programmed for a scanning between 192 and 798 nm at a speed of 1 Hz with a bandwidth of 5 nm. The detection was monitored using three individual channels (280, 320, and 360 nm) at a speed of 10 Hz with a bandwidth of 11 nm. The auto sampler’s temperature was set at 12 °C and the injection volume applied was 20 µL. The mobile phase consisted of water:formic acid (99.5%:0.5%) as eluent A and acetonitrile:formic acid (99.5%:0.5%) as eluent B at a flow rate of 0.30 mL/min. All solvents were filtered through a 0.22 µm PVDF membrane (Millipore, Billerica, MA, USA) prior to analysis. The system was run with the following gradient program: 0–10 min from 99 to 95% A; 10–30 min from 95 to 82% A; 30–44 min from 82 to 64% A; 44–64 min at 64% A; 64–90 min from 64 to 10% A; 90–100 min at 10% A; 100–101 min from 10 to 95% A; 101–120 min at 95% A; and finally returning to the initial conditions. The quantification of the phenolic compounds was performed by a calibration curve (0.78–100 ppm) obtained with gallic acid, chlorogenic acid, and quercetin-3-hexoside. The data acquisition system was the Chromeleon version 7.0 (Waltham, MA, USA) for DAD.

### 2.7. Total Phenolic Content

The total phenolic content (TPC) of the extracts was determined according to the Folin–Ciocalteu’s colorimetric method [29,30]. Briefly, 230 µL of milli-Q water, 10 µL of the extract, and 15 µL (0.25 N) of Folin–Ciocalteu’s reagent were added in the microplate and mixed at room temperature for 3 min. After, 45 µL of sodium carbonate solution (solution 35%) was added and the microplate was incubated for 1 h at room temperature in the dark. The absorbance of the samples was measured at 765 nm on a microplate spectrophotometer (Epoch2, Biotek (Winooski, VT, USA)) with the Gen5 3.02 data analysis software spectrophotometer. Gallic acid (1000 mg/L) was used as the standard for the calibration curve and the results were expressed as gallic acid equivalents (mg GAE/g). All measurements were performed in triplicates for each sample analyzed.

### 2.8. Antioxidant Activity

#### 2.8.1. Oxygen Radical Absorbance Capacity (ORAC) Assay

The ORAC assay was performed following a modified method [31] as described by Serra and collaborators (2011) [32] using a microplate fluorescent reader (FL800 Bio-Tek Instruments, Winooski, VT, USA). This assay assessed the ability of the antioxidant species in the sample to inhibit the oxidation of fluorescein (3 × 10^−4^ mM) catalyzed by peroxyl radicals generated from AAPH. Trolox was used as a reference standard and the results were expressed as Trolox equivalent antioxidant capacity (TEAC) per gram of the plant (µmol TEAC/g). Experiments were performed in triplicates for each sample analyzed.

#### 2.8.2. Hydroxyl Radical Scavenging Capacity (HOSC) Assay

The HOSC assay was based on the previous reported method [33] using the FL800 microplate fluorescence reader (FL800 Bio-Tek Instruments, Winooski, VT, USA). This assay measured the hydroxyl radical scavenging capacity of a sample using fluorescein (9.96 × 10^−8^ M) as a probe and a classic Fenton reaction with FeCl_3_ (3.42 mM) and H_2_O_2_ (0.20 M) as a source of hydroxyl radicals. Samples were analyzed in triplicates and results were expressed as Trolox equivalents antioxidant capacity (TEAC) per gram of the plant (μmol TEAC/g).

### 2.9. Antihypertensive Activity Assay

The antihypertensive activity of *S. ramosissima* extracts was evaluated using an angiotensin-converting enzyme (ACE) activity assay kit (Sigma-Aldrich, Saint Louis, MO, USA). This kit provides a simple, quick, sensitive, and direct procedure for measuring ACE levels to screen for ACE inhibitors and this assay is based on the cleavage of a synthetic fluorogenic peptide. Briefly, 10 µL of extract and 40 µL of ACE was added to a 96-well black microplate and incubated at 37 °C for 5 min to allow for contact between the enzyme and the inhibitor. Then, 50 µL of the substrate (ACE fluorogenic) were added and the fluorescence was read every minute for 5 min. A standard curve (0.1 to 0.8 nmol) was used for the quantification of the fluorescent product formed and the percentage of inhibition was calculated according to the manufacturer’s protocol (CS0002, Sigma-Aldrich). Lisinopril (Sigma-Aldrich, Darmstadt, Germany) was also used as a positive control. The IC_50_ value corresponds to the needed amount of extract to inhibit 50% of the ACE. The range of concentrations tested was between 500 and 31.25 mg/mL (59 to 3.68 mg/mL dw) for the extract from the fresh plant, and between 100 and 3.125 mg/mL for the extracts from the dried plants.

### 2.10. Cell-Based Assays

#### 2.10.1. Cell Culture

Human colon cancer cell lines, namely HT29 and Caco-2 cells, were obtained from the American Type Culture Collection (ATCC, Manassas, VA, USA) and Deutsche Sammlung von Microorganismen und Zellkulturen (Barunshweig, Germany), respectively. Both cell lines were grown in RPMI 1640 medium (Gibco, Carlsbad, CA, USA) supplemented with 10% (*v*/*v*) of heat-inactivated sterile filtered fetal bovine serum (FBS; Biowest, Riverside, CA, USA). For Caco-2 cells, its culture medium was supplemented with 1% (*v*/*v*) of PenStrep (Gibco, Carlsbad, CA, USA). Stock cells were maintained as monolayers in 75 cm^2^ culture flasks and incubated at 37 °C with 5% CO_2_ in a humidified atmosphere.

#### 2.10.2. Cytotoxicity Assay in Caco-2 Cells

Cytotoxicity assays were performed using confluent and non-differentiated Caco-2 cells. Briefly, cells were seeded at a density of 2 × 10^4^ cells/well in 96-well plates and allowed to grow for 7 days with medium renewal every 48 h [34]. At day 7, (complete confluence), Caco-2 cells were incubated with different concentrations of extracts, diluted in culture medium (RPMI medium with 0.5% FBS (Gibco, Carlsbad, CA, USA)). The range of concentrations tested was 31–500 mg/mL (3.7–59 mg/mL dw) for the extract from the fresh plant and 3.1–100 mg/mL for the extracts from the dried plants. The control of the solvent (50% of EtOH:H_2_O, *v*/*v*) diluted in the culture medium (RPMI medium with 0.5% FBS) was also tested. After 24 h of incubation at 37 °C with 5% CO_2_ in a humidified atmosphere, the cell viability was assessed using the CellTiter 96^®^ AQueous One Solution Cell Proliferation Assay (Promega, Madison, WI, USA) containing the MTS reagent according to manufacturer’s instructions. Absorbance was measured at 490 nm using a microplate spectrophotometer (Epoch 2, BioTek Instruments, Inc. (Winooski, VT, USA)). Results were expressed in terms of the percentage (%) of cellular viability relative to control (cells growth in culture medium only). At least three independent experiments were performed in triplicates.

#### 2.10.3. Antiproliferative Assay in HT29 Cells

The antiproliferative effect of plants was evaluated by testing their capacity in inhibiting the proliferation of HT29 cells as described previously, a cell line widely used as a model of in vitro colorectal cancer [32,35]. Briefly, cells were cultured in 96-well microplates at a density of 1 × 10^4^ cells/well. After 24 h of incubation at 37 °C in 5% CO_2_, the medium of each well was replaced by a medium containing the extracts prepared in the EtOH solution (EtOH:H_2_O, 50:50, *v*/*v*) and diluted in culture medium (RPMI medium with 0.5% FBS (Gibco, Carlsbad, CA, USA)). A control of the solvent (50% of EtOH:H_2_O, *v*/*v*) diluted in culture medium (RPMI with 0.5% FBS) was also tested. The range of concentrations tested was 31 to 500 mg/mL (59 to 3.68 mg/mL dw) for the extract from the fresh plant and 100 to 3.125 mg/mL for the extracts from the dried plants. A control of the solvent (50% of EtOH:H_2_O, *v*/*v*) diluted in culture medium (RPMI medium with 0.5% FBS) was also tested. Cells were incubated for 24 h with concentrations of the extracts, and after 24 h, the medium was removed and the cell viability was determined by MTS reagent as reported in the cytotoxicity tests. Results were expressed in terms of the percentage (%) of cellular viability relative to the control (cells growth in culture medium only). The amount of sample necessary to decrease 50% of the cellular viability, the EC_50_ (effective dose), was calculated. Experiments were performed in triplicates using at least three independent assays.

### 2.11. Sensory Analysis

The sensorial testing aimed to assess the acceptance and purchase intention of consumers for ketchup samples prepared with dried *S. ramosissima*. Two ketchup formulations were tested containing different amounts of oven-dried *S. ramosissima* as a salt substitute (an alternative to sodium chloride): (I) 2.2% (0.91 g of salt per 100 g of product) and (II) 3.0% (1.38 g of salt per 100 g of product). The samples were produced by Mendes Gonçalves S.A. (Golegã, Portugal) by homogenizing the tomato products with *S. ramosissima* and other natural ingredients. The acceptability testing was conducted with 102 consumers. The participants filled out a questionnaire assessing consumption information and food preferences. The consumers received the samples to evaluate attributes including appearance, aroma, and taste as well as their overall liking. Consumers rated the sensory characteristics of the products on a nine-point hedonic scale: 1 = “disliked extremely”, 2 = “disliked very much”, 3 = “disliked moderately”, 4 = “disliked slightly”, 5 = “neither liked nor disliked”, 6 = “liked slightly”, 7 = “liked moderately”, 8 = “liked very much”, and 9 = “liked extremely”. The purchase intention was also evaluated by participants, so they chose the option that best fitted their intention (“would not buy”, “would maybe buy”, and “would buy”). Samples were coded with 3-digit numbers and sample presentation was counterbalanced over the entire test to avoid order effects. Each sample (10 mL) was served in a plastic cup (30 mL) with a mini plastic spoon. Participants were instructed to drink water before and after each tasting.

### 2.12. Statistical Analysis

**Figure 1:**
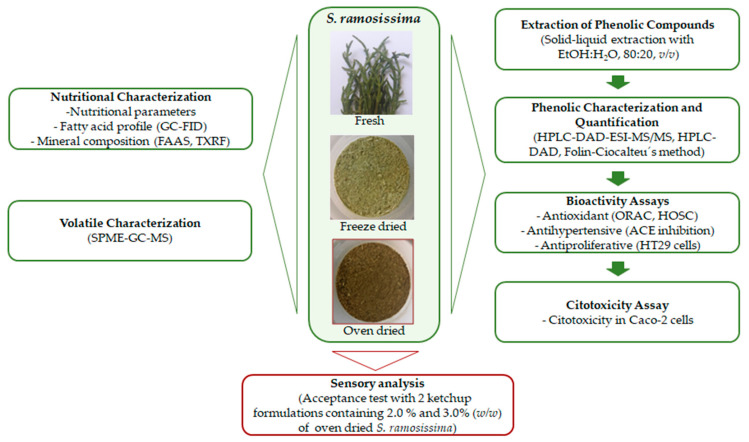
Scheme of the analyses and bioactivity assays of the fresh, oven, and freeze-dried *S. ramosissima*.

Results from chemical analysis were expressed as average ± standard deviation and statistical analysis was performed using GraphPad Prism 9.4 software (GraphPad Software, Inc., La Jolla, CA, USA) for one way analysis of variance (ANOVA), Tukey’s Test, and *t*-Test (*p* < 0.05). The correlation between TPC and antioxidant activity was determined with Pearson’s correlation coefficient test, considering a confidence level of 95% (*p* < 0.05). For antihypertensive and antiproliferative assays, the IC_50_ and EC_50_ values were calculated using non-linear regression (dose-response inhibition). The scheme of the analyses and bioactivity assays is shown in Figure 1.

## 3. Results and Discussion

### 3.1. Nutritional Characterization

In Table 1, the nutritional composition of fresh and dried *S. ramosissima* samples are presented. The results obtained for the fresh plant are in accordance with data already reported in the literature [10]. For all the parameters presented in Table 1, there were differences between the fresh (values reported as dried weight) and dried plants.

Steam succulent halophyte plants have a high percentage of water in their composition, with values higher than 84% [7,10,36]. The freeze-drying process removed the water content from plant matrix more efficiently than the oven-dried process (3.20% and 7.66%, respectively). The long time required for oven-drying at 70 °C (72 h) seals the surface capillaries of the samples, reducing the water releasing from the matrix [37]. The freeze-drying process allows to remove the water from the frozen plant using both primary (unbound water removal) and secondary (bound water removal) drying, thus producing the driest end-product [38].

The content of ashes and the total fat, carbohydrates, salt, and energy value did not display significant differences between both drying processes. However, freeze-dried *S. ramosissima* showed a higher protein content (11.00 g/100 g dw) than the oven-dried plant (8.48 g/100 g dw). Freeze-drying inhibited the degradation of protein, while heat drying processing lead to protein degradation and denaturation [39].

The total dietary fiber content of *S. ramosissima* increased with the oven-drying process at 70 °C and these results are in accordance to others observed in halophytes [40,41] and in fruits and vegetables. This [41,42] may be due to a modification in the structure of carbohydrates, which enhanced a structural rearrangement of the insoluble polysaccharides that might result in an increased quantification of total dietary fiber [42].

The main fatty acids present in fresh *S. ramosssima* are palmitic, linoleic, and linolenic acids, with a predominant amount of polyunsaturated fatty acids (PUFA) (58.40 g/100 g), mainly linolenic acid (33.50 g/100 g) (Table 1), and results are in accordance to data previously published [7,43,44]. The PUFA n-6/PUFA n-3 ratio was 0.72 in the lipophilic fraction of fresh *S. ramosissima* and corresponded, in this study, to the ratio of linoleic/linolenic acids. This ratio is important considering a n-6/n-3 ratio lower than 5 is associated with a significant decreased in the risk of cardiovascular, inflammatory, and autoimmune diseases, contributing to a more anti-inflammatory state [45,46].

For the dried plants, the *S. ramosissima* processed by freeze-drying presented the highest amount of PUFA (68.8 g/100 g) and the lowest amount of saturated fatty acids (SFA) (28.1 g/100 g), while the oven-dried plant presented the lowest PUFA value (52.7 g/100 g) and the highest SFA value (42.5 g/100 g). The linoleic and linolenic acids’ content for the oven-dried plant (23.8 g/100 g and 27.90 g/100 g, respectively) is lower compared to the freeze-dried plant (28.0 g/100 g and 40.2% g/100 g, respectively). Linoleic and linolenic acids’ content is known to be decreased by thermo-oxidation when exposed to a temperature at 70 °C [47].

The mineral composition of halophytes plants is known to depend on the plant’s growth zone as different amounts of the nutrients can be present in different soils. In Table 2, the values obtained for the fresh and dried plants studied are summarized. Sodium is known to be the most abundant macro-element in halophyte plants, including the *Salicornia* species, and its concentration increases with the increase of the cultivation media’s salinity [10,43].

The mineral composition of oven-dried *S. ramosissima* was lower than the one previously reported for the same species collected in a different region in Portugal, which was dried in an oven at 40 °C [7]. The sodium, potassium, zinc, and copper contents from *S. ramosissima* were not significantly affected by the drying process used but a reduction in the levels of iron and calcium occurred when the plant was dried using the oven process (Table 2). Similar results were reported when *Sarcocormia fruticosa* [48] was oven-dried.

The genus *Salicornia* is defined as a metal accumulator plant that is capable of accumulating and tolerating high pollutant concentrations from salt marshes [5,49], including heavy metals, leading to potential impacts on human health and safety [24]. Therefore, according to the Recommendation (EU) 2018/464/2018, it is necessary to monitor the heavy metals and iodine and establish their maximum limit levels in seaweed and halophyte plants. Regarding heavy metals, the fresh *S. ramosissima* presented levels of lead and cadmium below 0.3 and 0.2 mg/kg fw, respectively, notably the maximum content allowed by Regulation (EU) number 1881/2006 for leaf vegetables. Similarly, arsenic levels in the fresh and dried *S. ramosissima* are lower than the maximum levels allowed in certain foods by Commission Regulation (EU) number 2015/1006 (0.2 mg/kg fw). Selenium, iodine, and heavy metals such as Cd and Hg were below the detection limit of the method of analysis in the dried plants analyzed (Table 2).

Oven-drying has been accepted as an important method of preservation, especially when taking into consideration the lower cost when compared to the freeze-drying process. Results presented showed that the oven drying process had a low impact on the nutritional composition of *S. ramosissima*, suggesting that this process could be a good option to extend its shelf-life. Furthermore, the oven-drying process may be use for large-scale industrial production as well as for micro-scale production. Overall, the drying process in the oven at 70 °C displayed a reduction on the protein, the Fe and Ca contents, and increased the dietary fiber and saturated fatty acids contents when compared with the freeze-drying process.

### 3.2. Volatile Compounds Profile

The volatile composition from fresh and dried *S. ramosissima* was studied by GC-MS. For the identification, the mass spectra comparison with the mass spectra bank, the retention time, and the Linear Retention Index (LRI) were determined. Compounds were classified by their chemical classes and odor descriptions according to the literature (Appendix A, Table A1). In order to compare contents, the percentage of the area of peaks compared to the total area of the chromatogram was measured.

**Figure 2:**
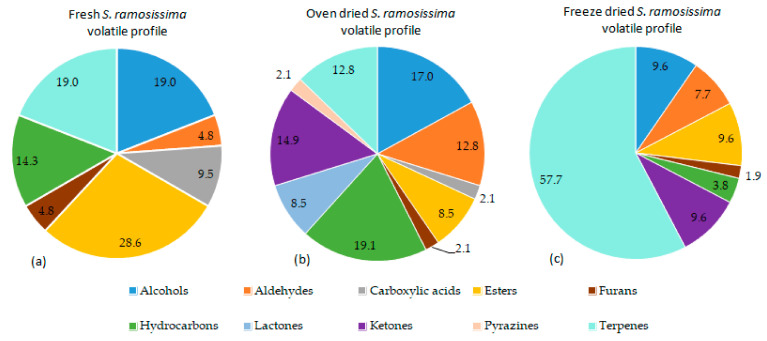
Pie charts showing the chemical classes profile of volatile compounds of the *S. ramosissima*. (**a**) Volatile compounds profile of fresh plant; (**b**) volatile compounds profile of oven-dried plant; and (**c**) volatile compounds profile of freeze-dried plant.

Many chemical classes of compounds were identified in both the dried and fresh *S. ramosissima*, such as alcohols, aldehydes, carboxylic acids, esteres, furans, hydrocarbons, ketones, pyrazines, and terpenes, as summarized in Figure 2.

In the fresh *S. ramosissima* samples, two peaks of high intensity were identified, which corresponded to 3-hexen-1-ol (47.95%) and 1-hexanol (47.82%). Both alcohols are known to be responsible for green-type odors and 3-hexen-1-ol has also been described as a seaweed odor [50,51]. In addition, hexanal (0.26%), ethyl tiglate (1.51%), 3-hexen-1-ol acetate (0.33%), and methyl and ethyl benzoate (0.54% and 0.21%, respectively) are responsible for odors such as green, herbal, fruity, and floral [52,53].

For the oven-dried *S. ramosissima*, the main volatile compounds detected were hexanal (34.16%), 3-hexen-1-ol (1.80%), 2-methylbutanoic acid (7.84%), heptanal (5.14%), 1-octen-3-ol (3.0%), 6-methyl-5-hepten-2-one (3.92%), *p*-cymene (3.08%), limonene (10.18%), 3,4-dimethylcyclohexanol (7.31%), β-cyclocitral (1.97%), and 3,5-octadien-2-one (1.19%). In general, linear and branched chain aldehydes contribute with herbaceous and grassy-green aromas [54], namely hexanal, which is responsible for herbal and grassy-green odors [52,53]. Compounds such as 2-methylbutanoic acid, heptanal, 1-octen-3-ol, and 3,5-octadien-2-one are described as responsible for some off-odors including sour, penetrating oil-like, mushroom-like, and marine odors, respectively, but their contribution to the aroma depends on their limit of detection [50,53,55,56].

The major compounds identified in the freeze-dried *S. ramosissima* include β-myrcene (2.26%), α-phellandrene (8.45%), *p*-cymene (8.80%), 1,8-cineole (38.73%), β-thujone (7.21%), α-thujone (2.31%), camphor (4.80%), and isobornyl acetate (8.17%). These terpenes and esters are responsible for odors described as herbaceous, fresh, and green [57,58,59,60,61], specifically 1,8-cineole that has an odor description of eucalyptus, spicy, and pepper. In addition, (Z,Z)-3,6-nonadien-1-ol was identified in the freeze-dried *S. ramosissima* (0.30%), which has been described as contributing to the odor of marine and seaweed of *Mertensia maritima* L. [50].

Both drying methods induced changes in the volatile profile compared to the fresh *S. ramosissima*. For the oven-dried plant, a loss of compounds such as esters, terpenes, and alcohols was observed and has been justified by the effect of the temperature [62]. Additionally, the formation of lactones, pyrazines, and ketones, and the increase of hydrocarbons and lactones, was observed. Pyrazines and ketones can be produced during the oven-drying process from free amino acids and monosaccharides by the Maillard reaction through Strecker degradation, and they contribute to the roast-like aroma and buttery flavor [63]. Conversely, lactones can be generated by the degradation of phenolic acids during the oven-drying, such as chlorogenic acids [64].

The oven-drying process significantly decreased the contribution of terpenes (17.02%) and increased aldehydes (12.77%) compared to the freeze-drying process. A previous study showed that alcohols such as 3-hexen-1-ol and 3,6-nonadien-1-ol detected by GC−olfactometry are responsible for the fresh and marine odors in the halophyte plant *Mertensia maritima* L. [50]. Oven-dried *S. ramosissima* is characterized by a green and herbal aroma, therefore the seaweed odor characteristic is less intensive compared to the fresh plant due to reduction of 3-hexen-1-ol [50].

The freeze-drying process is less aggressive than the oven-drying used due to the low temperature and oxygen content [65,66,67]. Results show that in the freeze-dried plant, there was an increase in the contribution of the terpenes class from 19.0 to 57.7% and these compounds are usually responsible for aromas described as fresh, eucalyptus, and camphoraceous. Terpenes were also the most represented volatiles in freeze-dried sea fennel (*Crithmum maritimum* L.), specifically limonene, γ-terpinene, α-pinene, *p*-cymene, sabinene, myrcene, α-thujene, camphene, β-thujene, *α*-terpinene, (Z)-β-ocimene, and terpinolene, which have also been identified in *S. ramosissima* [68].

### 3.3. Phytochemical Characterization and Biological Activities

#### 3.3.1. Identification and Quantification of Phenolic Compounds

The phenolic compounds of the fresh, oven-dried, and freeze-dried *S. ramosissima* were tentatively identified by LC-DAD-ESI-MS/MS with ESI negative ionization mode. Ionization conditions in the mass spectrometer were optimized in order to detect the *m*/*z* corresponding to the precursor ions. Table 3 shows a list of 29 compounds tentatively identified, including their retention time (t_R_), UV absorption maxima, precursor ion, the MS/MS product ions, and the bibliographic references to support the putative identification. The compounds were numbered by their elution order as most of them were not found in all plant extracts. Among the identified phenolic compounds, 15 hydroxycinnamic acids (neochlorogenic, chlorogenic, *p*-coumaric, ferulic, and caffeic glycosides acids) and nine flavonoids (quercetin glycosides and apigenin glycoside) were identified.

Some organic acids were identified in *S. ramosissima* extracts such as malic (peak 2) and quinic (peak 3) acids with percursor ions [M–H]^−^ at *m*/*z* 133 and 191, respectively, and product ions according to the literature [69,70]. Compounds 4 and 10 were identified as neochlorogenic acid (5-*O*-caffeoylquinic acid) and chlorogenic acid (3-*O*-caffeoylquinic acid), respectively. These phenolic acids are characterized by a percursor ion at *m*/*z* 353 and product ions at *m*/*z* 191, 179, and 135 [71,72,73]. In this study, chlorogenic acids are distinguished due to their different retention times and the difference in product ions relative intensities. The product ions at *m*/*z* 179 and 135 are in general more intense in neochlorogenic acid [72,73]. In addition, the chlorogenic acid standard was analyzed and this identification was confirmed (Table 4).

Chlorogenic acid is found naturally in various plants, fruits, and vegetables such as coffee beans, apples, and blueberries, and its bioavailability depends largely on its metabolism by the gut microflora, as it is hydrolyzed into caffeic acid and quinic acid [74]. Chlorogenic acids and their derivates have health beneficial effects including neuroprotective, anti-inflammatory, cardiovascular protective, hepatoprotective, antihypertensive, glucose and lipid metabolism regulatory, and anticarcinogenic effects [12,75,76,77].

Caffeoylquinic dimers were also identified, such as 3,5-dicaffeoylquinic (peak 24) and 4,5-dicaffeoylquinic acids (peak 25) with a percursor ion [M–H]^−^ at *m*/*z* 515 and product ion characteristics of caffeoylquinic, quinic, and caffeic acids (*m*/*z* 353, 191, and 179, respectively) [70,78]. Previous studies reported antihypertensive and hypoglycemic activity for dicaffeoylquinic acids [79,80,81].

Flavonoids such as quercetin-3-glucoside (peak 21) and quercetin-malonyglucoside (peak 23) were identified. Quercetin 3-glucoside presented a precursor ion [M–H]^−^ at *m*/*z* 463 and a product ion at *m*/*z* 301 (aglycone quercetin), in line with the cleavage of a hexosyl residue (162 amu) [82]. Quercetin-malonyglucoside displayed a precursor ion [M-H]^−^ at *m*/*z* 549 and a product ion at *m*/*z* 505 (loss of CO_2_, 44 amu), *m*/*z* 463 (loss of CH_2_O, 42 amu), and 301 (162 amu), corresponding to the cleavage of a malonyhexose (248 amu) [83,84]. Quercetin glycosides are known to present antioxidant, anti-carcinogenic, antiviral, antibacterial, and anti-inflammatory activities [85,86].

**Table 3:** Phenolic compounds putatively identified in the fresh, oven-dried, and freeze-dried extracts from *S. ramosissima*.

Peak	t_r_ (min.) ^a^	λ_max_ (nm)	Precursor Ion [M-H]^−^ *m*/*z*	Product Ion *m*/*z*	Tentative Identification	Extracts ^b^	References
1	7.14	301	215	191, 179	quinic acid derivative	FH	[69,70]
2	8.58	275	133	133, 115, 113, 71	malic acid	OD	[70,71,87]
3	9.36	260	191	111, 87, 85	quinic acid	FH, OD, FD	[69,70]
4	29.03	300,325	353	191, 179, 135	neochlorogenic acid	FH, OD, FD	[71,72,88]
5	32.77	280,317	285	153, 152, 108, 109	protocatechuic-acid-arabinoside	FH	[69,89]
6	33.83	275	305	305, 225, 97, 59	gallocatechin	FH, FD	[90]
7	34.85	284,318	355	137, 93	salicylic acid derivative	FH, OD	[91]
8	35.38	281,332	355	273, 253, 191, 173	hydrocaffeoylquinic acid	FD	[15]
9	35.63	274, 310	163	119	*p*-coumaric acid	FH, FD	[78,92]
10	36.58	300,327	353	191, 179	chlorogenic acid *	FH, OD, FD	[71,72,88]
11	38.63	267,345	193	134, 161, 178	ferulic acid	FH, OD, FD	[69,88]
12	40.26	269,332	355	193, 178, 161, 134	ferulic acid-glucoside	FH, OD, FD	[69,87,88]
13	40.69	268,337	303	303, 97	dihydroquercetin (taxifolin)	FH, OD, FD	[93,94]
14	42.40	312	337	191, 173, 163	*p*-coumaroylquinic acid (isomer 1)	FH, OD, FD	[95,96]
15	43.88	-	371	249, 121, 113	saccharide	FH, OD, FD	[97]
16	44.60	311	337	215, 191, 173, 163	*p*-coumaroylquinic acid (isomer 2)	OD	[95,96]
17	45.49	256,336	319	295, 294, 187, 97	n.i.	FH, FD	
18	46.34	270,354	609	301, 151	quercetin-rhamnosyl-hexoside	FH, OD, FD	[98]
19	46.50	300	449	253, 118	*p*-coumaric acid benzyl ester derivative	OD	[99]
20	47.10	274,335	519	315, 301, 300, 299	quercetin-methyl-ether derivative (isomer 1)	FH, FD	[100]
21	47.64	255,352	463	463, 301, 300	quercetin 3-glucoside*	FH, OD, FD	[15,71,78]
22	48.18	284,329	517	517, 355, 179, 135	caffeic acid-glucuronide-glucoside (isomer 1)	FD	[88,101]
23	48.62	251,358	549	505, 463, 301, 300	quercetin-malonyglucoside	FH, OD, FD	[71,84,102]
24	49.37	302,332	515	353, 325, 191, 179	3,5-dicaffeoylquinic acid	FH, OD, FD	[70,96]
25	50.38	302,329	515	479, 353, 191, 179, 173	4,5-dicaffeoylquinic acid	FH, OD, FD	[70,78,96]
26	50.94	266,331	517	355, 179, 135	caffeoyl-hydrocaffeoylquinic acid	FH, OD, FD	[15]
27	51.66	268,346	519	519, 350, 315, 300	quercetin-methyl-ether derivative (isomer 2)	FH, OD, FD	[100]
28	52.33	328	563	503, 473, 459, 443, 383, 353	apigenin-6-arabinosyl-8-glucoside (isoschaftoside)	FH	[94,103]
29	59.90	287	953	953, 767, 575, 285	kaempferol derivative	FH, FD	[71,104]

^a^ Mean value for retention times obtained from the analysis of fresh, oven-dried and freeze-dried *S. ramosissima* extracts. ^b^ Extracts: FH, fresh; OD, oven-dried; and FD, freeze-dried. * Phenolic compound identified with standard. n.i. means not identified.The phenolic composition of *S. ramosissima* samples was in compliance with the literature [15,105]. The occurrence of quinic acid, *p*-coumaric and ferulic acid, 3-*O*-caffeoylquinic acid, and quercetin-3-*O*-glucoside in aqueous extracts [105], and quercetin-glucoside, hydrocaffeoylquinic, caffeoylquinic, dihydrocaffeoyl quinic, caffeoyl-hydrocaffeoylquinic acid, and dicaffeoyl quinic acids in an ethyl acetate fraction isolated from *S. ramosissima* were already described [15]. Quercetin-glucoside, caffeoylquinic acid, and other hydroxycinnamic acid derivatives were also reported in other *Salicornia* species [19,106,107].

In the dried plants, it was possible to putatively identify some compounds that had not been identified in the fresh plant, such as a second isomer of *p*-coumaroylquinic acid and a *p*-coumaric acid benzyl ester derivative in the oven-dried plant, and hydrocaffeoylquinic acid and an isomer of caffeic acid-glucuronide-glucoside in the freeze-dried plant. Furthermore, quercetin 3-glucoside, quercetin-malonyhexoside, 3,5-dicaffeoylquinic acid, and 4,5-dicaffeoylquinic acid showed a higher concentration in the freeze-dried plant than in the fresh plant (Table 4) and it could be attributed to the higher extraction efficiency. The freeze-drying process is responsible for the higher porosity (80% to 90%) of the freeze-dried plant material compared to the material obtained by the convective-drying method [108], increasing solvent diffusion through dried tissue, and consequently resulting in the transfer of phenolic compounds to the solvent [109].

Despite the freeze-drying process generating a significant increase in the amount of dicaffeoylquinic acids and quercetin glucosides compared to the fresh plant (µg/g dw), for protocatechuic-arabinoside acid, ferulic acid, dihydroquercetin, *p*-coumaroylquinic acid (isomer 1), a quercetin-methyl-ether derivative, and a kaempferol derivative, a decrease was observed, as shown in Table 4. This effect may be related to oxidation, the most important biochemical process that occurs during the processing of the plants, which starts as soon as the integrity of the cell is broken [110], namely during the grinding process for sample preparation. The total flavonoids and total hydroxycinnamic acids in the freeze-dried plant were 5151.65 µg/g dw and 1512.32 µg/g dw, while in the oven-dried plant they were lower (2393.97 µg/g dw and 1211.73 µg/g dw, respectively), as can be seen in Table 4. These results show that the oven-drying process generated a reduction of 54% in the total flavonoids and 20% in the total hydroxycinnamic acids compared to freeze-dried plant.

Similar to our results, studies [80,111,112] have showed that the freeze-drying process preserves the bioactive compounds and retains a higher amount of polyphenols in herbs, while the oven-drying process causes major loss of these compounds, especially with drying temperatures higher than 60 °C. The freeze-drying process is able to preserve the polyphenol content as it prevents its thermal and oxidative degradation, and limits enzymatic reactions from polyphenol oxidase, lipoxygenase, and peroxidase [113,114,115]. Oven-drying methods have demonstrated to have a significant impact on the flavonoid content. Flavonoids are negatively affected by hot air-drying, which are degraded proportionally to an increase in the temperature [113,116].

In general, the results show that the oven-drying process affects the phenolic composition, reducing the quercetin glycosides, but the chlorogenic acids and their derivatives are minimally affected. In terms of phenolic composition, the freeze-drying method is less aggressive than the oven-drying method.

**Table 4:** Quantification of phenolic compounds in the fresh, oven-dried, and freeze-dried extracts of *S. ramosissima*.

Peak	Quantified Compounds ^ab^	Fresh (µg/g fw)	Fresh (µg/g dw)^c^	Oven-Dried (µg/g dw)	Freeze-Dried (µg/g dw)
3	quinic acid ***	9.33 ± 2.54	79.11 ± 21.52 ^B^	117.4 ± 1.28 ^aA^	58.67 ± 1.51 ^bB^
4	neochlorogenic acid *	18.03 ± 4.40	152.80 ± 37.27 ^A^	136.12 ± 2.51 ^aA^	106.81 ± 3.06 ^bA^
5	protocatechuic-arabinoside acid *	3.00 ± 0.10	25.43 ± 0.87	-	-
6	gallocatechin **	5.25 ± 0.78	44.54 ± 6.58 ^a^	-	25.52 ± 0.44 ^b^
7	salicylic acid derivative *	2.54 ± 0.01	21.53 ± 0.08 ^a^	13.74 ± 0.30 ^b^	-
8	hydrocaffeoylquinic acid *	-	-	-	12.82 ± 0.04
*9*	*p*-coumaric acid *	2.75 ± 0.04	23.33 ± 0.37 ^a^	-	19.00 ± 0.26 ^a^
10	chlorogenic acid *	53.19 ± 13.59	450.76 ± 115.17 ^A^	318.74 ± 11.11 ^bA^	421.91 ± 13.08 ^aA^
11	ferulic acid *	4.21 ± 0.40	35.72 ± 3.38 ^A^	24.01 ± 0.13 ^bB^	26.58 ± 0.39 ^aB^
12	ferulic-glucoside acid *	5.98 ± 0.40	50.69 ± 3.36 ^A^	21.23 ± 9.35 ^aB^	46.92 ± 2.15 ^aA^
13	dihydroquercetin **	2.86 ± 0.08	24.21 ± 0.65 ^A^	18.58 ± 1.62 ^aB^	14.52 ± 0.13 ^aB^
14	*p*-coumaroylquinic acid (isomer 1) *	2.72 ± 0.09	2.08 ± 0.74 ^A^	15.48 ± 0.06 ^aB^	13.77 ± 0.04 ^bB^
16	*p*-coumaroylquinic acid (isomer 2) *	-	-	24.63 ± 0.74	-
18	quercetin-rhamnosyl-hexoside **	7.65 ± 1.13	64.81 ± 9.62 ^A^	52.90 ± 1.34 ^bA^	74.49 ± 2.31 ^aA^
19	*p*-coumaric acid benzyl ester derivative *	-	-	72.51 ± 2.22	-
20	quercetin-methyl-ether derivative (isomer 1) **	13.60 ± 1.09	115.26 ± 9.20 ^a^	-	35.21 ± 22.13 ^b^
21	quercetin 3-glucoside **	53.53 ± 8.11	453.66 ± 68.76 ^B^	688.38 ± 16.48 ^aA^	636.02 ± 19.47 ^aA^
22	caffeic acid-glucuronide-glucoside (isomer 1) *	-	-	-	53.40 ± 1.64
23	quercetin-malonyglucoside **	309.39 ± 60.74	2621.99 ± 514.72 ^B^	1578.0 ± 30.0 ^bB^	4202.52 ± 48.09 ^aA^
24	3,5-dicaffeoylquinic acid *	25.83 ± 0.05	218.87 ± 0.40 ^B^	223.38 ± 9.27 ^bB^	434.88 ± 13.95 ^aA^
25	4,5-dicaffeoylquinic acid *	11.75 ± 0.533	99.58 ± 4.52 ^B^	223.22 ± 9.67 ^aA^	213.09 ± 5.97 ^aA^
26	caffeoyl-hydrocaffeoylquinic acid *	9.72 ± 3.13	82.40 ± 26.59 ^B^	138.67 ± 7.37 ^aAB^	163.14 ± 4.64 ^aA^
27	quercetin-methyl-ether derivative (isomer 2) **	13.36 ± 2.70	113.23 ± 22.91 ^A^	56.11 ± 1.43 ^bB^	101.18 ± 2.26 ^aAB^
28	apigenin-6-arabinosyl-8-glucoside (isoschaftoside) **	3.82 ± 0.11	32.38 ± 0.96	-	-
29	kaempferol derivative **	10.90 ± 2.09	92.42 ± 17.71 ^a^	-	62.19 ± 1.75 ^b^
	Total Flavonoids	420.36	3562.50	2393.97	5151.65
	Total Hydroxycinnamic	139.72	1163.19	1211.73	1512.32

^a^ Data are expressed as means values ± standard deviation (*n* = 2). ^b^ The lower-case letters (a and b) correspond to the significant difference by the unpaired *t*-test (*p* < 0.05). The upper-case letters (A,B) indicate the significant differences by Tukey’s test (*p* < 0.05). ^c^ The values were converted from fresh weight to dried weight according to the moisture value. Phenolic compounds were identified with standard. (*) Chlorogenic acid and derivatives were quantified as a chlorogenic acid equivalent. (**) Quercetin-3-glucose and quercetin glycosylated derivatives were quantified as a quercetin-3-glucose equivalent. (***) Quinic acid was quantified as a gallic acid equivalent.

#### 3.3.2. Bioactivity: Antioxidant, Antihypertensive, and Antiproliferative Effects

As mentioned above, the drying method is considered the most common technique for the postharvest preservation of herbs and edible plants; however, this technique is reported to have an influence on the content of bioactive compounds in several food matrices [112], including *S. ramosissima*. In Table 5, the impact of the drying process on the total phenolic content (TPC) and bioactivity of *S. ramossima*, namely antioxidant and antihypertensive activities, is described.

The TPC value of fresh *S. ramosissima* was 1.02 mg GAE/g fw and these values are in the same range as previously described for fresh *Salicornia genus*, between 1.05 and 1.53 mg GAE/g fw [43]. This value is above the lower limit of other condiments rated as rich in phenolic compounds (<1.0 mg GAE/g fw), such as celery (0.31), garlic (1.01), onion yellow (0.95), capsicum green (0.59), leek (0.85) [117], and coriander (0.60) [118].

Results showed that a lower TPC value was observed in the oven-dried extract (7.41 mg GAE/g) than in the fresh and freeze-dried plant extract (8.64 and 9.74 mg GAE/g dw, respectively). The TPC values from the freeze-dried extract are also in accordance to data already reported (7.03 to 12.09 mg GAE/g dw) [10] and values for other species of freeze-dried *Salicornia*, namely *Salicornia europaea* (5.6 to 9.30 mg GAE/g dw) [119]. In contrast, studies on oven-dried *S. ramosissima* reported higher TPC values than the one reported in this work (27.44 to 33.00 mg GAE/g dw) [7,15]. This difference can be due to several factors related to the culture conditions of the fresh plant including the salt stress conditions and environmental changes [10,36]. Halophytes live in extremely harsh environments with high salinities and UV radiation, and these stressful conditions lead to the production of secondary metabolites such as the phenolic compounds in different concentrations [7].

The phenolic content of dried plants has high positive significant correlations with their antioxidant activity (average *r* > 0.9741). Correlation between the TPC and ORAC assay was 0.985, while between TPC and HOSC was 0.962. The freeze-drying plant presented a significantly higher value of the ORAC compared to the fresh plant (202.10 µmol TEAC/g dw) and a similar value in the HOSC assay (220.21 µmol TEAC/g dw). This result can be explained by the highest concentrations observed of the flavonoids in the freeze-dried plant [120]. Flavonoids are generally more capable of inactivating peroxyl radicals than the small phenolic antioxidants, whereas hydroxycinnamic acids are very effective in the inactivation of hydroxyl radicals [121].

This study showed that there are differences among the drying processes, which can be explained by the reduction of the phenolic profile of the oven-dried plant compared to freeze-dried (Table 4). The oven-drying process presented an average reduction of 31% in antioxidant activity probably due to the reduction of quercetin glycosides. The reactions involved in the decrease of phenolics compounds with increasing drying temperature (40–70 °C) are associated to enzymatic and non-enzymatic oxidation reactions [21,122]. Similarly to our results, studies also showed that the oven-drying process of *S. fruticosa* exposed to a high temperature (70 °C) for a long time contributed to the reduction of the polyphenols content [21] and the loss of antioxidant properties [48].

The antihypertensive activity of *S. ramosissima* samples was evaluated through of the ACE inhibition assay. The ACE inhibitors are commonly used in therapy for the treatment of hypertension and cardiovascular diseases [123,124]. IC_50_ values obtained for the dried plants were significantly different from the IC_50_ value of the fresh plant. However, IC_50_ values of dried plants did not show significant differences between them, indicating that the oven-drying process does not have a significant impact on the compounds that are responsible for the inhibition of the ACE enzyme.

**Figure 3:**
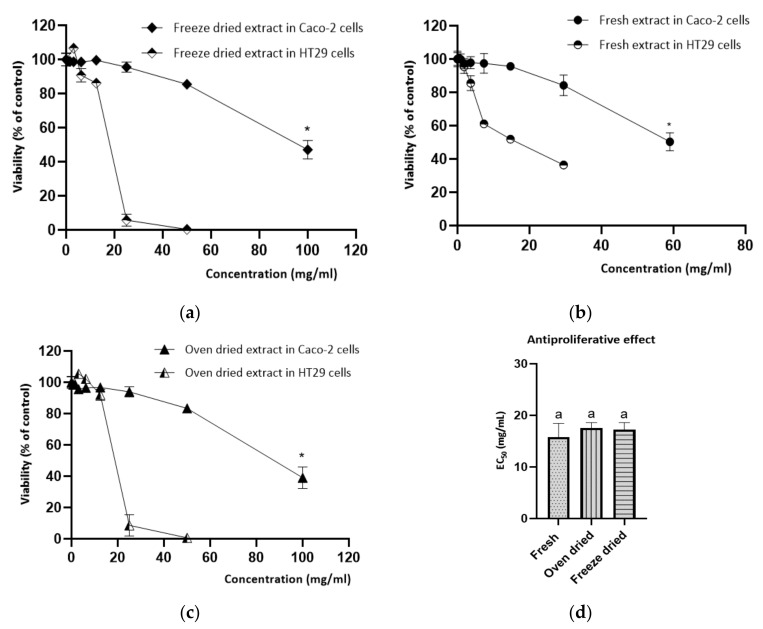
Fresh, oven-dried, and freeze-dried extracts from *S. ramosissima* exerted antiproliferative effects in a dose-dependent manner after exposition for 24 h. Dose-response curves of the antiproliferative effect induced by the (**a**) freeze-dried extract, (**b**) oven-dried extract, and (**c**) fresh extract. (**d**) EC_50_ values of the antiproliferative response induced by the fresh (EC_50_ =15.82 ± 2.65 mg/mL), oven-dried (EC_50_ = 17.56 ± 1.05 mg/mL), and freeze-dried (EC_50_ = 17.24 ± 1.36 mg/mL) extracts in HT29 cells (cell model of CRC) did not show significant differences. Results shown are means of at least three independent experiments performed in triplicates ± SD. (*)represent the existence of statistical difference between that dose and the previous one, using one-way ANOVA for multiple comparisons by Tukey’s test (*p* < 0.05).

In the same manner, both dried plants demonstrated similar antiproliferative effects in colorectal cancer cells (HT29) (Figure 3). Fresh and dried extracts had the ability to impair HT29 cell proliferation in a dose-dependent manner (Figure 3a–c), with an EC_50_ value of 15.82 mg/mL dw, 17.56 mg/mL, and 17.24 mg/mL for the fresh, oven-dried and freeze-dried plants, respectively (Figure 3d). It is important to mention that the EC_50_ values determined in this work did not present cytotoxic effects in confluent Caco-2 cells, a cell model widely used to evaluate the effect of chemicals and food bioactive compounds in intestinal function.

The similar bioactive response in terms of the antiproliferative and anti-hyperglycemic effects among both drying samples could be attributed to their similar composition in bioactive compounds, namely quercetin-3-*O*-glucoside, neochlorogenic acid, ferulic acid-glucoside, caffeic acid-glucuronide-glucoside, and 3,5-dicaffeoylquinic acid (Table 4). Accordingly, it has been reported that quercetin glycosides have a significant impact on ACE inhibition, in which quercetin-3-*O*-glucoside is the most effective ACE inhibitor among the flavonoids’ glycosides [125,126]. Quercetin glucosides also have an antiproliferative activity in human cancer cells with inhibitory effects on proliferation of HT29 cells [14,127]. Similarly, caffeoylquinic acids are able to reduce HT29 cell viability, promoting specific changes in the cell cycle, and increase the apoptosis rate [11,128,129], and their derivatives are found to be effective as natural ACE inhibitors [126,130,131].

In addition, the synergistic effects between quercetin glycosides and caffeoylquinic acids in the dried extracts from *S. ramosissima* may also have contributed to similar antihypertensive and antiproliferative activities. Phenolic extracts from halophyte plants and their isolated polyphenols have been studied in a number of cancer cell lines and antihypertensive models [132,133,134,135,136]. Extracts from halophytes, such as *Salicornia herbacea* seed cells, showed potent antioxidant activity and selective cytotoxic effects against HT29 and HCT116 human colon adenocarcinoma [132]. In addition, *Artemisia scoparia*, *Cocos nucifera*, and *Tribulus terrestris* showed ACE inhibitory activity [135,136,137,138,139].

### 3.4. Sensorial Analysis: Consumer Acceptance of Oven-Dried S. ramosissima

Aimed at evaluating the acceptance of *S. ramosissima* as a natural salt substitute, a sensory test with a ketchup formulated with dried *S. ramosissima* at 2.2% (sample 2.2% DS) and 3.0% *w/w* (sample 3.0% DS) was carried out with 102 volunteers (69% women and 31% men, aged between 29 and 59 years old). The information obtained in the questionnaires about each consumer is shown in Supplementary Materials Table S1. For this experiment, the oven-dried plant was selected as it presents several advantages over the freeze-dried sample, including (i) the reduction of alcohols, which could impact the consumer acceptability due to the association with marine odor, and (ii) low processing costs, which is more attractive to food industry applications. Additionally, this sample also showed similar bioactive effects as the freeze-dried plant, indicating that the lower phenolic composition did not impact its antiproliferative and antihypertensive potential.

**Figure 4:**
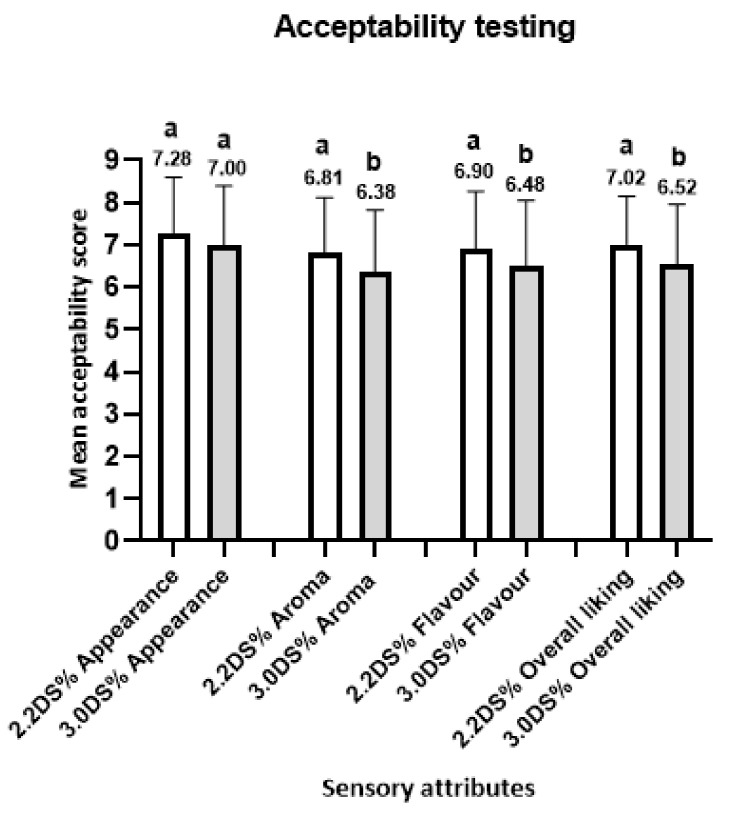
Evaluation mean scores for sensory attributes of the ketchups with 2.2% oven-dried *S. ramosissima* (2.2DS%) and 3.0% oven-dried *S. ramosissima* (3.0DS%) (*n* = 102 consumers). Each sensory attribute was evaluated using a nine-point hedonic scale where 1 = “disliked extremely” and 9 = “liked extremely”. The letters (a and b) correspond to the statistical analysis performed to calculate the existence of a significant difference (*p* < 0.05) between each sample according to the unpaired *t*-test.

Consumers evaluated the sensory characteristics of the two ketchups, including appearance, aroma, flavor, and overall liking. The mean scores for the ketchup sensory attributes are presented in Figure 4.

Consumer acceptability scores for overall liking, aroma, and flavor were significantly different (*p* > 0.05) across the two ketchup formulations as the sample 2.2% DS showed higher consumer acceptability compared to sample 3.0% DS. In contrast, the consumer acceptability scores for appearance were not significantly different between the two ketchups. The results of the evaluation indicate a good consumer acceptance for the ketchup with the lower addition of the oven-dried *S. ramosissima* (sample 2.2% DS) and, consequently, a product with less salt content (0.91% of salt) (see Supplementary Materials Table S2). Although the consumption of ketchup is mostly associated with unhealthy eating, the addition of the dried *S. ramosissima* as a table salt substitute in ketchup becomes a potential ingredient to be considered, which can be applied for the development of low sodium products.

## 4. Conclusions

This study contributed by evaluating the effect of two drying processes in the preservation of the nutritional composition and bioactive compounds present in *S. ramosissima*. The results generated herein demostrated that both drying processes, namely oven-drying and freeze-drying, have an impact on the nutritional composition of *S. ramosissima*, as well as in the phytochemical content and antioxidant capacity, which are significantly reduced during the oven-drying of the plant compared to the freeze-drying process. Neverthless, antiproliferative and antihypertensive activities of the dried plants are not dependent on the type of drying process, meaning that other compounds present in theses plants may be more important for these activities.

During the oven-dried process, the volatile composition was also affected, leading to a decrease of compounds such as alcohols. Some of these compounds have been associated with marine odors and may affect the consumer acceptability of food products formulated with *S. ramosissima* as a natural ingredient.

These results indicate that the dried *S. ramosissima* could be an adequate option for use in the food and gourmet cuisine industry to replace table salt, even in the dried form, as they can have high amounts of NaCl and also phytochemicals that are important for their contribution in the prevention of diseases. The extracts from the analyzed plants also demonstrate potencial bioactivity and more research should be done concerning not only the other phytochemicals that may be present in these plants, but also the study of the amounts of plants that should be used in order be a salt substitute ingredient in a healthy diet.

## Figures and Tables

**Table 1:** Nutritional parameters and fatty acids of the fresh, oven, and freeze-dried *S. ramosissima*.

Sample	Fresh		Oven-Dried	Freeze-Dried
	(g/100 g fw)	(g/100 g dw) ^d^	(g/100 g dw)	(g/100 g dw)
*Nutritional parameters ^ab^*				
Moisture	88.20 ± 0.88	-	7.66 ± 0.08 ^a^	3.20 ± 0.03 ^b^
Ashes	5.91 ± 0.24	50.08 ± 0.34 ^A^	41.00 ± 1.64 ^aB^	44.20 ± 1.77 ^aB^
Protein	1.59 ± 0.06	13.47 ± 0.51 ^A^	8.48 ± 0.34 ^bC^	11.00 ± 0.44 ^aB^
Total fat	0.40 ± 0.01	3.39 ± 0.03 ^A^	1.20 ± 0.01 ^aC^	1.80 ± 0.02 ^aB^
Carbohydrates	2.90 ± 0.12	24.58 ± 1.02 ^A^	12.70 ± 0.51 ^aC^	12.90 ± 0.52 ^aB^
Total dietary fiber	1.00 ± 0.03	8.47 ± 0.26 ^C^	29.00 ± 0.87 ^aA^	26.90 ± 0.81 ^bB^
Energy value (kcal/100 g)	23.60 ± 0.94	200.01± 8.00 ^A^	153.50 ± 6.1 ^aC^	165.6 ± 6.6 ^aB^
Salt	5.62 ± 0.73	47.63 ± 6.20 ^A^	36.8 ± 4.8 ^aAB^	28.5 ± 3.7 ^aB^
*Fatty acids profile ^abc^*				
Palmitic acid (C16:0)	24.00 ± 0.01	24.00 ± 0.01 ^B^	26.80 ± 0.01 ^aA^	19.80 ± 0.01 ^bC^
Stearic acid (C18:0)	2.00 ± 0.01	2.00 ± 0.01 ^A^	1.90 ± 0.01 ^aB^	1.30 ± 0.01 ^bC^
Oleic acid (C18:1)	5.00 ± 0.01	5.00 ± 0.01 ^A^	3.50 ± 0.01 ^aB^	1.60 ± 0.01 ^bC^
Linoleic acid (C18:2)	24.10 ± 0.01	24.10 ± 0.01 ^B^	23.80 ± 0.01 ^bC^	28.10 ± 0.01 ^aA^
Linolenic acid (C18:3)	33.50 ± 0.01	33.50 ± 0.01 ^B^	27.90 ± 0.01 ^bC^	40.40 ± 0.01 ^aA^
Arachidic acid (C20:0)	0.90 ± 0.01	0.90 ± 0.01 ^B^	1.20 ± 0.01 ^aA^	0.80 ± 0.01 ^bC^
Eicosenoic acid (C20:1)	<0.05 *	<0.05 *	0.10 ± 0.01 ^a^	0.10 ± 0.01 ^a^
Behenic acid (C22:0)	1.40 ± 0.01	1.40 ± 0.01 ^C^	4.20 ± 0.01 ^aA^	2.10 ± 0.01 ^bB^
Lignoceric acid (C24:0)	1.70 ± 0.01	1.70 ± 0.01 ^C^	2.90 ± 0.01 ^aA^	2.50 ± 0.01 ^bB^
SFA	34.30 ± 0.01	34.30 ± 0.01 ^B^	42.50 ± 0.01 ^aA^	28.10 ± 0.01 ^bC^
MUFA	7.30 ± 0.01	7.30 ± 0.01 ^A^	4.80 ± 0.01 ^aB^	3.10 ± 0.01 ^bC^
PUFA	58.40 ± 0.01	58.40 ± 0.01 ^B^	52.70 ± 0.01 ^bC^	68.80 ± 0.01 ^aA^
PUFA n-6/PUFA n-3	0.72 ± 0.01	0.72 ± 0.01 ^A^	1.03 ± 0.01 ^bB^	0.70 ± 0.01 ^aA^
PUFA/SFA	1.70 ± 0.01	1.70 ± 0.01 ^B^	1.24 ± 0.01 ^bC^	2.45 ± 0.01 ^aA^

* (LOQ < 0.05 g/100 g). Abbreviations: SFA, total saturated fatty acids; MUFA, total monounsaturated fatty acids; and PUFA, total polyunsaturated fatty acids. ^a^ Data are expressed as average values ± standard deviation (*n* = 3). ^b^ The lower-case letters (a and b) correspond to the significant difference between the drying methods by unpaired *t*-test (*p* < 0.05). The upper-case letters (A–C) indicate the significant differences between the fresh plant and the drying process by Tukey’s test (*p* < 0.05). ^c^ Data are expressed in percentages of total methyl esters ± standard deviation (*n* = 2). ^d^ The values were converted from fresh weight to dried weight according to the moisture content.

**Table 2:** Mineral composition of the fresh, oven-dried, and freeze-dried *S. ramosissima*.

Sample	Fresh (fw)	Oven-Dried (dw)	Freeze-Dried (dw)
*Macro-elements (mg/g) ^ab^*			
Sodium (Na)	22.50 ± 2.90	147.01 ± 14.85 ^a^	114.02 ± 19.11 ^a^
Calcium (Ca)	0.32 ± 0.03	0.237 ± 0.003 ^b^	0.312 ± 0.002 ^a^
Potassium (K)	1.05 ± 0.22	0.409 ± 0.003 ^a^	0.411 ± 0.002 ^a^
*Micro-elements (* *µg/g) ^ab^*			
Iron (Fe)	37.00 ± 0.5	45.025 ± 0.347 ^b^	73.145 ± 0.442 ^a^
Manganese (Mn)	2.30 ± 0.32	4.581 ± 0.104 ^a^	2.830 ± 0.056 ^b^
Zinc (Zn)	6.20 ± 0.90	14.249 ± 0.143 ^a^	13.727 ± 0.094 ^a^
Copper (Cu)	1.20 ± 0.10	2.507 ± 0.045 ^a^	2.270 ± 0.037 ^a^
Selenium (Se)	<0.02 *	<0.02 *	<0.02 *
Iodine (I)	<0.13 *	<0.03 *	<0.03 *
*Heavy metals (µg/g) ^ab^*			
Arsenic (As)	0.15 ± 0.04	0.264 ± 0.015 ^a^	0.273 ± 0.019 ^a^
Cadmium (Cd)	0.010± 0.001	<0.10 *	<0.10 *
Mercury (Hg)	<0.03 *	<0.03 *	<0.03 *
Lead (Pb)	0.18 ± 0.02	1.525 ± 0.028 ^a^	1.508 ± 0.037 ^a^

* (LOD, limit of detection µg/g). ^a^ Data are expressed as means values ± standard deviation (*n* = 3). ^b^ The letters correspond to the statistical analysis performed to calculate the existence of a significant difference between both drying methods by the unpaired *t*-test (*p* < 0.05).

**Table 5:** Total phenolic content and both antioxidant and antihypertensive activities of extracts from the fresh, oven-dried, and freeze-dried *S. ramosissima*.

Processing Plant	Fresh (fw) ^a^	Fresh (dw) ^abc^	Oven-Dried (dw) ^ab^	Freeze-Dried (dw) ^ab^
TPC (mg GAE/g)	1.02 ± 0.04	8.64 ± 0.34^A^	7.41 ± 0.29 ^bB^	9.74 ± 0.88 ^aA^
*Antioxidant activity*				
ORAC (µmol TEAC/g)	23.85 ± 3.04	202.10 ± 25.81 ^B^	291.10 ± 17.95 ^bB^	418.81 ± 54.01 ^aA^
HOSC (µmol TEAC/g)	26.06 ± 2.29	220.21 ± 15.51 ^A^	147.21 ± 12.44 ^bB^	237.20 ± 12.02 ^aA^
*Antihypertensive activity*				
ACE inhibition (IC_50_ = mg/mL)	95.61 ± 14.13	12.60 ± 1.73 ^A^	24.56 ± 1.74 ^aB^	18.96 ± 0.62 ^aB^

^a^ Data are expressed as means values ± standard deviation (*n* = 3). ^b^ The lower-case letters (a and b) correspond to the statistical analysis performed to calculate the existence of a significant difference (*p* < 0.05) between both drying methods by the unpaired *t-*test. The upper-case letters (A,B) correspond to the statistical analysis performed to calculate the existence of a significant difference (*p* < 0.05) by Tukey’s test. ^c^ The values were converted from fresh weight to dried weight according to the moisture value.

## Data Availability

The data supporting the findings of this study are available within the article and its Supplementary Materials.

## Supplementary Materials

The following are available online at https://www.mdpi.com/article/10.3390/antiox10081312/s1.

## Appendix A

**Table A1:** Characterization parameters (retention time (tr), % of total peak area, identification, chemical classes, odor, calculated retention index) of volatile compounds identified in fresh, oven dried and freeze dried *S. ramosissima* using SPME-GC–MS and the bibliographic references to support the identification.

Peak	t_r_ (min.) ^a^	Area% Fresh	Area % oven Dried	Area % Freeze Dried	Compounds	Chemical Classes	Odor Description ^b^	Calc. LRI ^c^	Lit. LRI	References
1	5.012	0.26	34.16	1.30	hexanal	Aldehyde	herbal, grassy, green	800	801	[35,50,140,141]
2	6.700	0.46	-	-	1-methoxy-3-hexene	Hydrocarbon	not found	847	826	[142]
3	6.962	-	-	0.68	2-hexenal	Aldehyde	floral, herbal	853	850	[140,141]
4	7.103	47.95	1.80	-	3-hexen-1-ol	Alcohol	green, marine, seaweed	857	852	[50,52,143]
5	7.635	-	-	0.04	3-methyl-1-pentanol	Alcohol	fruity, floral	871	852	[144,145]
6	7.653	47.82	-	-	1-hexanol	Alcohol	woody, sweet, green, fruity	867	872	[53,141,146]
7	7.684	-	7.84	-	2-methylbutanoic acid ethyl ester	Ester	cheesy, sour	872	876	[55]
8	8.722	-	5.14	0.10	heptanal	Aldehyde	penetrating oily, harsh	901	902	[35,53,147]
9	9.079	-	1.29	-	butyrolactone	Lactone	cheesy, burnt sugar; buttery	911	915	[53,146,147,148]
10	9.278	-	0.17	-	2-3-dimethylpyrazine	Pyrazine	nutty, cocoa-like	916	918	[53,149]
11	9.569	-	-	0.05	alpha-thujene	Terpene	herbal, green, weak earthy	924	924	[60,150,151]
12	9.786	-	-	0.88	alpha-pinene	Terpene	oily, green	930	933	[58,151]
13	10.260	1.51	-	-	ethyl tiglate	Ester	fruity	943	939	[152]
14	10.274	-	-	1.09	camphene	Terpene	sweet	943	947	[58,151,153]
15	10.616	-	0.25	-	*γ*-valerolactone	Lactone	sweet, herbaceous	953	950	[154,155]
16	10.792	-	1.39	-	benzaldehyde	Aldehyde	hazelnut, roasty	958	962	[156,157]
17	11.288	-	-	1.37	beta-pinene	Terpene	Woody, green, pine-like	971	979	[150,153,158]
18	11.416	-	0.31	-	3,5,5-trimethyl-2-hexene	Hydrocarbon	not found	975	977	[159]
19	11.637	-	3.00	0.01	1-octen-3-ol	Alcohol	mushroom	981	980	[50,56,150]
20	11.848	-	3.92	0.05	6-methyl-5-hepten-2-one	Ketone	banana-like	987	988	[143,160]
21	11.973	0.00	0.00	2.26	beta-myrcene	Terpene	herbaceous, sweet	990	991	[58,150,151]
22	12.004	0.07	1.14	-	2-pentylfuran	Furan	floral, fruit	991	990	[154,161]
23	12.164	0.07	0.60	-	hexanoic acid	Carboxylic acid	unpleasant, rancing, metallic	995	993	[162,163]
24	12.321	-	0.99	-	decane	Hydrocarbon	gasoline-like, fishy	1000	1000	[164,165]
25	12.329	-	-	8.45	alpha-phellandrene	Terpene	fresh, green	1000	1005	[59,146,153]
26	12.372	-	0.16	-	octanal	Aldehyde	fruity, green, citrus	1001	1005	[146,159,166]
27	12.663	0.33	-	-	3-hexen-1-ol acetate	Ester	fruity, floral	1010	1007	[140,141]
28	12.793	-	-	0.24	alpha-terpinene	Terpene	resinous	1014	1017	[59,150]
29	12.834	-	0.39	-	2-methylpentyl formate	Ester	not found	1015	-	
30	12.917	0.11	-	-	hexyl acetate	Ester	fruit, herb	1017	1019	[145,167]
31	13.083	0.02	3.08	8.80	*p*-cymene	Terpene	green, fruity, aromatic	1022	1027	[58,158,168]
32	13.225	0.16	10.18	-	limonene	Terpene	pine/chemical, floral/fresh	1027	1029	[56,156]
33	13.306	-	-	38.73	1,8-cineole	Terpene	eucalyptus, spicy, pepper	1029	1032	[57,156,169]
34	13.353		1.14	-	2-ethyl-1-hexanol	Alcohol	green, flowery, green cucumber	1031	1029	[141,163,170]
35	13.517	0.08	-	-	2-hexenoic acid	Carboxylic acid	not found	1035	-	
36	13.643	-	0.29	-	3-octen-2-one	Ketone	rose	1039	1040	[161,171,172]
37	13.719	-	0.16	-	phenylacetaldehyde	Aldehyde	lilac, flora	1041	1043	[55,156]
38	13.808	0.06	-	-	benzyl alcohol	Alcohol	floral, fruity, rose	1044	1045	[173]
39	14.225	-	-	0.29	gamma-terpinene	Terpene	green, woody	1057	1059	[55,58,172]
40	14.502	-	-	0.13	trans-sabinene hydrate	Terpene	spicy, weak fruity	1065	1060	[60,150]
41	14.731	-	1.19	0.04	3,5-octadien-2-one	Ketone	green, marine, grass, fatty	1072	1098	[50,56]
42	15.194	-	-	0.37	alpha-terpinolene	Terpene	woody, herbaceous	1086	1086	[55,59,107]
43	15.503	-	0.30	-	linalool oxide	Terpene	woody	1095	1092	[168,174]
44	15.508	0.54	-	-	methyl benzoate	Ester	eucalyptus, phenolic, wood	1095	1094	[175,176]
45	15.633	-	-	0.48	linalool	Terpene	pleasant scent, floral	1099	1097	[141,158]
46	15.640	-	0.14	-	2,6,11-trimethyldodecane	Hydrocarbon	not found	1099	1102	[177]
47	15.724	-	-	7.21	beta-thujone	Terpene	camphoraceous-herbal	1102	1119	[60]
48	15.789	-	7.31	-	3,4-dimethylcyclohexanol	Alcohol	not found	1104	1103	[178]
49	16.069	-	-	2.31	alpha-thujone	Terpene	warm-herbal, minty	1113	1114	[60,179]
50	16.165	-	0.09	-	4-hexen-1-ol, 2-ethenyl-2,5-dimethyl-	Alcohol	not found	1116	-	
51	16.233	0.11	-	-	phenylethyl alcohol	Alcohol	sweet, perfume, floral, bee wax	1118	1117	[50,143]
52	16.566	-	-	0.14	1,3,8-*p*-menthatriene	Terpene	green, cucumber, floral	1129	1130	[56,144]
53	16.903	-	-	4.80	camphor	Terpene	camphoraceous, fresh	1140	1141	[55,60]
54	17.429	-	-	0.08	trans-pinocamphone	Terpene	cedar, camphoreous, woody	1157	1157	[180,181]
55	17.627	-	-	0.18	borneol	Terpene	camphoraceous, earthy	1163	1165	[60,169]
56	17.674	-	-	0.27	(Z,Z)-3,6-nonadien-1-ol	Alcohol	green, marine, seaweed	1165	1165	[50,182]
57	17.847	-	-	0.04	cis-pinocamphone	Terpene	cedar, camphoreous, woody	1171	1172	[181]
58	17.925	0.21	-	-	ethyl benzoate	Ester	flowery	1173	1172	[156,183]
59	17.983	-	-	0.21	terpinen-4-ol	Terpene	green, fruity, citrus-like	1175	1178	[58]
60	18.436	-	0.32	0.43	alpha-terpineol	Terpene	minty, fresh vegetable, green	1190	1189	[163,184]
61	18.566	-	-	0.11	cis-dihydrocarvone	Terpene	cooling, fresh, minty	1194	1193	[185,186]
62	18.659	-	1.16	-	safranal	Terpene	saffron-like	1197	1197	[187,188]
63	18.749	0.02	1.02	-	dodecane	Hydrocarbon	alkane-like, chemical	1201	1200	[159,189]
64	18.892	-	0.42	-	decanal	Aldehyde	soapy, chemical	1205	1205	[156,190]
65	18.910	-	-	0.25	verbenone	Ketone	sweet, floral, camphor-like	1206	1204	[146,191]
66	19.056	-	-	0.34	4,7-dimethyl-benzofuran	Furan	smoke, moss, spicy	1211	1220	[56,164]
67	19.223	-	-	0.07	cuminaldehyde	Aldehyde	sweet, fresh	1217	1207	[58,192]
68	19.314	0.08	1.97	-	beta-cyclocitral	Terpene	sweet-tobacco, grape	1220	1220	[156,193]
69	19.697	-	-	0.93	thymol methyl ether	Terpene	oregano-like, smoky-like, woody	1233	1233	[150,194]
70	19.782	-	-	0.22	octyl-acetate	Ester	fruity, herbal	1236	1222	[145,195]
71	19.957	-	-	0.31	isothymol methyl ether	Terpene	burnt, smoky-like, woody	1242	1244	[196]
72	20.116	-	-	3.29	1,2-Dimethyl-4-isobutylbenzene	Hydrocarbon	not found	1248	1240	[197]
73	20.150	-	0.39	-	1-(4-ethylphenyl)-ethanone	Ketone	not found	1249	1231	[198]
74	21.037	-	-	0.30	decanol	Alcohol	floral, spicy	1280	1280	[182,199]
75	21.051	0.01	0.16	-	4,6-dimethyldodecane	Hydrocarbon	not found	1281	1285	[200]
76	21.157	-	-	8.17	isobornyl acetate	Ester	herb, woody, sweet, minty	1284	1285	[61,169]
77	21.417	-	-	0.56	thymol	Terpene	thyme-like, spicy	1294	1293	[60,194]
78	21.620	-	0.97	0.10	2-undecanol	Alcohol	tallowy, soapy	1301	1287	[190,201]
79	21.730	-	0.64	-	2,4-diethyl-1-heptanol	Alcohol	not found	1305	1321	[202]
80	21.964	-	0.59	-	2-isopropyl-5-methyl-1-heptanol	Alcohol	not found	1314	1300	[203]
81	22.149	-	0.46	-	2,2,4,4,6,8,8-heptamethylnonane	Hydrocarbon	not found	1321	1321	
82	22.965	-	0.39	-	1-hydroxy-2,4,4-trimethyl-3-pentanyl 2-methylpropanoate	Ester	fruity	1351	-	[155]
83	23.416	-	-	0.14	(3-Isopropenyl-2-methylcyclopentyl)methyl acetate	Ester	not found	1368	-	
84	23.628	0.06	0.19	0.27	3-hydroxy-2,4,4-trimethylpentyl 2-methylpropanoate	Ester	apple, fresh, cucumber	1376	1376	[204,205]
85	24.238	-	0.75	-	tetradecane	Hydrocarbon	alkane-like, chemical	1399	1413	[189,206]
86	24.451	-	-	0.78	ethyl decanoate	Ester	fruity	1407	1407	[55,207]
87	24.730	-	-	0.11	trans-caryophyllene	Terpene	fresh, fruity, citrus	1418	1419	[58,59,184]
88	24.813	-	-	0.37	thymohydroquinone dimethyl ether	Terpene	earthy, moldy	1422	1426	[208,209]
89	25.048	-	-	0.24	γ-Elemene	Terpene	green, citrus, floral	1431	1434	[58,210]
90	25.228	-	-	0.05	alloaromadendrene	Terpene	woody	1438	1442	[211,212]
91	25.349	-	-	2.10	geranyl acetone	Ketone	fresh, green	1443	1449	[149,168]
92	25.603	-	0.24	0.15	(E)-nerylacetone	Ketone	rose, fresh, green, magnolia	1454	1452	[213,214]
93	25.810	-	0.24	-	γ-decalactone	Lactone	candy, sweet	1462	1467	[55,141,149,168]
94	26.350	-	0.52	-	4-(2,6,6-trimethylcyclohexa-1,3-dienyl)but-3-en-2-one	Ketone	not found	1483	1483	[215]
95	26.412	-	1.49	-	beta-ionone	Ketone	dry fruit, floral	1486	1486	[55,168]
96	26.644	-	0.22	-	pentadecane	Hydrocarbon	herbaceous	1495	1500	[58,168]
97	26.692	0.06	-	-	valencene	Terpene	fruity, flowery	1496	1496	[58,216,217]
98	27.094	-	0.19	-	2,4-di-tert-butylphenol	Hydrocarbon	not found	1513	1513	[218,219]
99	27.470	-	1.26	-	dihydroactinidiolide	Lactone	coumarin-like, musky	1529	1528	[220,221]
100	30.835	-	-	0.11	1-heptadecene	Hydrocarbon	earthy, moss	1677	1673	[157,222]

^a^ Average of the three retention times obtained for the chromatograms of the fresh, oven dried, and freeze dried *S. ramosissima*. ^b^ Descriptions taken from the literature. ^c^ Average of the three calculated linear retention indexes obtained for the chromatograms of the fresh, oven dried, and freeze dried *S. ramosissima.*
