# Supplementary material for: Impact of Drying Processes on the Nutritional Composition, Volatile Profile, Phytochemical Content and Bioactivity of Salicornia ramosissima J. Woods
Source: Antioxidants (Basel). 2021 Aug 20;10(8):1312. doi: 10.3390/antiox10081312 (PMC8389250; doi:10.3390/antiox10081312)
Supplement: Supplementary file 1 [file antioxidants-10-01312-s001.zip › antioxidants-1324028-supplementary.pdf]

antioxidants

Table S1. Characteristics of participants of the Acceptability testing of ketchups with oven dried *S. ramosissima.*

**Sensory Test (n = 102)**	***n***	**%**
Gender		
Male	32	31%
Female	70	69%
Ages		
[18-20]	2	2
[21-30]	51	50
[31-40]	26	25
[41-50]	12	12
[51-60]	11	11
Nationality		
Portugal	95	93
Other	7	7
Education		
High-school or less	12	12
Bachelor's degree	21	21
Master's degree	39	38
Doctor's degree	30	29
Ketchup consumption frequency		
Daily	0	0
Twice a week	3	3
Once a week	24	24
Once a month	35	34
Rarely	39	38
Never	1	1
Typical foods eaten with ketchup [a]		
French fries	92	35
Pork or other meat sandwiches	39	15
Other sandwiches	6	2
Pizza	13	5
Salad	2	1
Pasta	15	6
Hamburgers	80	31
Other	12	5

[a] The participant was allowed to select more than one option in this question, so the percentages were adjusted to the total number of responses.

Table S2. Nutritional parameters, and mineral and fatty acids composition of the ketchups with 2.2% dried *S. ramosissima* (DS) and 3.0% dried *S. ramosissima* used in sensorial analysis (Acceptability testing).

**Ketchup sample designation**	**2.2% DS**	**3.0% DS**
Nutritional composition (g/100g)		
Moisture	71.30 ± 0.71[a]	68.40 ± 0.68[b]
Ashes	1.86 ± 0.07[b]	2.34 ± 0.09[a]
Protein	1.68 ± 0.07[b]	2.00 ± 0.08[a]
Total fat	0.20 ± 0.01[a]	0.20 ± 0.01[a]
Carbohydrates	22.80 ± 0.91[a]	24.40 ± 0.98[a]
Total sugar	21.60 ± 3.24[a]	21.20 ± 3.18[a]
Total dietary fiber	2.20 ± 0.07[b]	2.70 ± 0.08[a]
Energy value (kcal/100g)	104.10 ± 4.16[a]	112.80 ± 4.51[a]
Chlorides	1.08 ± 0.10[b]	1.36 ± 0.15[a]
Salt	0.91 ± 0.12[b]	1.38 ± 0.18[a]
Fatty acids profile (g/100g) [c]		
Myristic acid (C14:0)	2,62 ± 0.01[a]	2,45 ± 0.01[b]
Palmitic acid (C16:0)	24,0 ± 0.01[a]	24,0 ± 0.01[a]
Stearic acid (C18:0)	3,83 ± 0.01[a]	3,73 ± 0.01[b]
Oleic acid (C18:1)	5,70 ± 0.01[a]	5,65 ± 0.01[a]
Linoleic acid (C18:2)	40,9 ± 0.01[a]	40,5 ± 0.01[a]
Linolenic acid (C18:3)	19,2 ± 0.01[b]	20,2 ± 0.01[a]
Arachidic acid (C20:0)	0,84 ± 0.01[a]	0,86 ± 0.01[a]
Arachidonic acid (C20:4)	0,38 ± 0.01[a]	0,24 ± 0.01[b]
Behenic acid (C22:0)	0,74 ± 0.01[b]	0,82 ± 0.01[a]
Lignoceric acid (C24:0)	0,76 ± 0.01[b]	0,87 ± 0.01[a]
SFA	33,5 ± 0.01[a]	33,0 ± 0.01[a]
MUFA	6,10 ± 0.01[a]	6,02 ± 0.01[a]
PUFA	60,5 ± 0.01[a]	61,0 ± 0.01[a]
Mineral composition (mg/100g)		
Sodium (Na)	364 ± 47.3[b]	550 ± 71.5[a]
Calcium (Ca)	21.40 ± 2.57[a]	27.30 ± 3.28[a]
Potassium (K)	330 ± 69.3[a]	370 ± 77.7[a]
Iron (Fe)	1.47 ± 0.21[a]	1.34 ± 0.19[a]
Magnesium (Mg)	39.90 ± 5.59[a]	50.40 ± 7.06[a]
Manganese (Mn)	0.28 ± 0.04[a]	0.35 ± 0.05[a]
Zinc (Zn)	0.22 ± 0.03[a]	0.28 ± 0.04[a]
Copper (Cu)	<0.05*	0.10 ± 0.01[a]

*(LOQ = 0.05 g/100g), SFA - total saturated fatty acids, MUFA - total monounsaturated fatty acids, PUFA - total polyunsaturated fatty acids. [a] Data are expressed as means values ± standard deviation (n = 3). [b] The letters correspond to the statistical analysis performed to calculate the existence of a significant difference ($p < 0.05$), between both drying methods, by unpaired t test. [c] Data are expressed in percentages of total methyl esters ± standard deviation (n=3).
